# Histidine *N*τ-methylation identified as a new posttranslational modification in histone H2A at His-82 and H3 at His-39

**DOI:** 10.1016/j.jbc.2023.105131

**Published:** 2023-08-04

**Authors:** Takahiro Hayashi, Hiroaki Daitoku, Toru Uetake, Koichiro Kako, Akiyoshi Fukamizu

**Affiliations:** 1 Doctoral Program in Life and Agricultural Sciences, Degree Programs in Life and Earth Sciences, Graduate School of Sciences and Technology, University of Tsukuba, Tsukuba, Ibaraki, Japan; 2 Life Science Center for Survival Dynamics, Tsukuba Advanced Research Alliance, University of Tsukuba, Tsukuba, Ibaraki, Japan; 3 Institute of Life and Environmental Sciences, University of Tsukuba, Tsukuba, Ibaraki, Japan; 4 International Institute for Integrative Sleep Medicine, University of Tsukuba, Tsukuba, Ibaraki, Japan; 5 AMED-CREST, Japan Agency for Medical Research and Development, Chiyoda-ku, Tokyo, Japan

**Keywords:** histone methylation, histidine, mass spectrometry, chromatography, S-adenosylmethionine

## Abstract

Histone posttranslational modifications play critical roles in a variety of eukaryotic cellular processes. In particular, methylation at lysine and arginine residues is an epigenetic mark that determines the chromatin state. In addition, histone “histidine” methylation was initially reported over 50 years ago; however, further studies in this area were not conducted, leaving a gap in our understanding. Here, we aimed to investigate the occurrence of histidine methylation in histone proteins using highly sensitive mass spectrometry. We found that acid hydrolysates of whole histone fraction from calf thymus contained *N*τ-methylhistidine, but not *N*π-methylhistidine. Both core and linker histones carried a *N*τ-methylhistidine modification, and methylation levels were relatively high in histone H3. Furthermore, through MALDI-TOF MS, we identified two histidine methylation sites at His-82 in the structured globular domain of histone H2A and His-39 in the N-terminal tail of histones H3. Importantly, these histidine methylation signals were also detected in histones purified from a human cell line HEK293T. Moreover, we revealed the overall methylation status of histone H3, suggesting that methylation is enriched primarily at lysine residues and to a lesser extent at arginine and histidine residues. Thus, our findings established histidine *N*τ-methylation as a new histone modification, which may serve as a chemical flag that mediates the epigenetic mark of adjacent residues of the N-terminal tail and the conformational properties of the globular domain.

In eukaryotes, genomic DNA is tightly packed into the dynamic structure of chromatin, nucleosomes being its basic units (1). Histones are the pivotal components of nucleosome particles, forming an octamer containing two copies each of the core histone proteins, H2A, H2B, H3, and H4, which contain reversible complexes with a 147-bp segment of DNA (2). Each globular histone protein has an intrinsic disordered region, the N-terminal tail, characterized by relatively high contents of the basic amino acids, lysine, and arginine residues. Histone N-terminal tails are targets for multiple covalent posttranslational modifications, such as acetylation, methylation, and phosphorylation, which coordinate to determine the chromatin state, thereby regulating gene expression (3, 4, 5). Among the histone modifications, methylation, the attachment of a methyl group (-CH_3_) to the side chains of lysine and arginine, represents a subtle but complex modification. It occurs in three distinct forms, mono-, di-, and tri-methylation of lysine and mono-, symmetric di-, and asymmetric di-methylation of arginine (6, 7). Growing evidence indicates that the individual methylation states of histone lysine or arginine serves as key epigenetic marks, with their combinatorial patterns playing leading roles in the histone code hypothesis (5, 8, 9).

In addition to lysine and arginine, protein methylation also occurs at another basic histidine residue, where a methyl group is added to the nitrogen at either position 1 (*N*π) or 3 (*N*τ) of the imidazole ring. Protein histidine methylation was first found in *N*τ-methylhistidine (also referred to as 3-methylhistidine) (τMH), a constituent of actin and myosin isolated from muscle tissue in 1967 (10). However, it required over 4 decades to discover the first histidine-specific protein methyltransferase Hpm1 in budding yeast. In 2010, Hpm1 was shown to catalyze the *N*τ-methylation of His-243 in the ribosomal protein Rpl3, consequently contributing to the assembly of the large ribosomal subunit and translational elongation fidelity (11, 12, 13, 14). Recently, mammalian SETD3 was identified to be responsible for the *N*τ-methylation of actin at His-73 and SETD3-deficient female mice were found to develop primary maternal dystocia (15, 16, 17). Meanwhile, we and another group have independently uncovered that mammalian METTL9 introduces *N*π-methylhistidine (1-methylhistidine) (πMH) at His-x-His (where x denotes a small side-chain residue) motif-containing proteins, including the proinflammatory protein S100A9 (18, 19). Thus, although histidine methylation had garnered attention as an early established and newly elucidated posttranslational modification, it remains poorly studied due to methodological limitations of histidine methylation analysis; for example, the isomers πMH and τMH have identical mass and cannot be distinguished using mass spectrometry (MS) alone (20).

Reviewing the literature of histone modification, the presence of histidine methylation in histone fractions from avian erythrocytes and human HeLa cells was suggested over 50 years ago (21, 22). Nevertheless, in contrast to the methylation on lysine and arginine, histone histidine methylation has not yet been firmly established. Therefore, in this study, we set out to determine whether histidine methylation occurs in histone using a unique method of analyzing methyl amino acids derived from hydrolysate of histone proteins combined with liquid chromatography coupled to tandem mass spectrometry (LC-MS/MS) (18, 23). We found that an unfractionated whole histone protein from the calf thymus contained τMH but not πMH. Both core and linker histones were subjected to histidine *N*τ-methylation, and histone H3 was highly methylated compared to other histones. Furthermore, MALDI-TOF MS analysis revealed that histones H2A and H3 were methylated at His-82 and His-39, respectively. Importantly, these two histidine residues were also methylated in histones purified from a human cell line HEK293T. The overall methylation status of histone H3 was disclosed, in which methylation was significantly enriched at lysine residues and clearly detectable at arginine and histidine residues. Our findings provided evidence that histones indeed carry a τMH modification and suggest that this methylation has additional implications for the histone code hypothesis.

## Results

### Whole histone from calf thymus contains τMH

**Figure 1:**
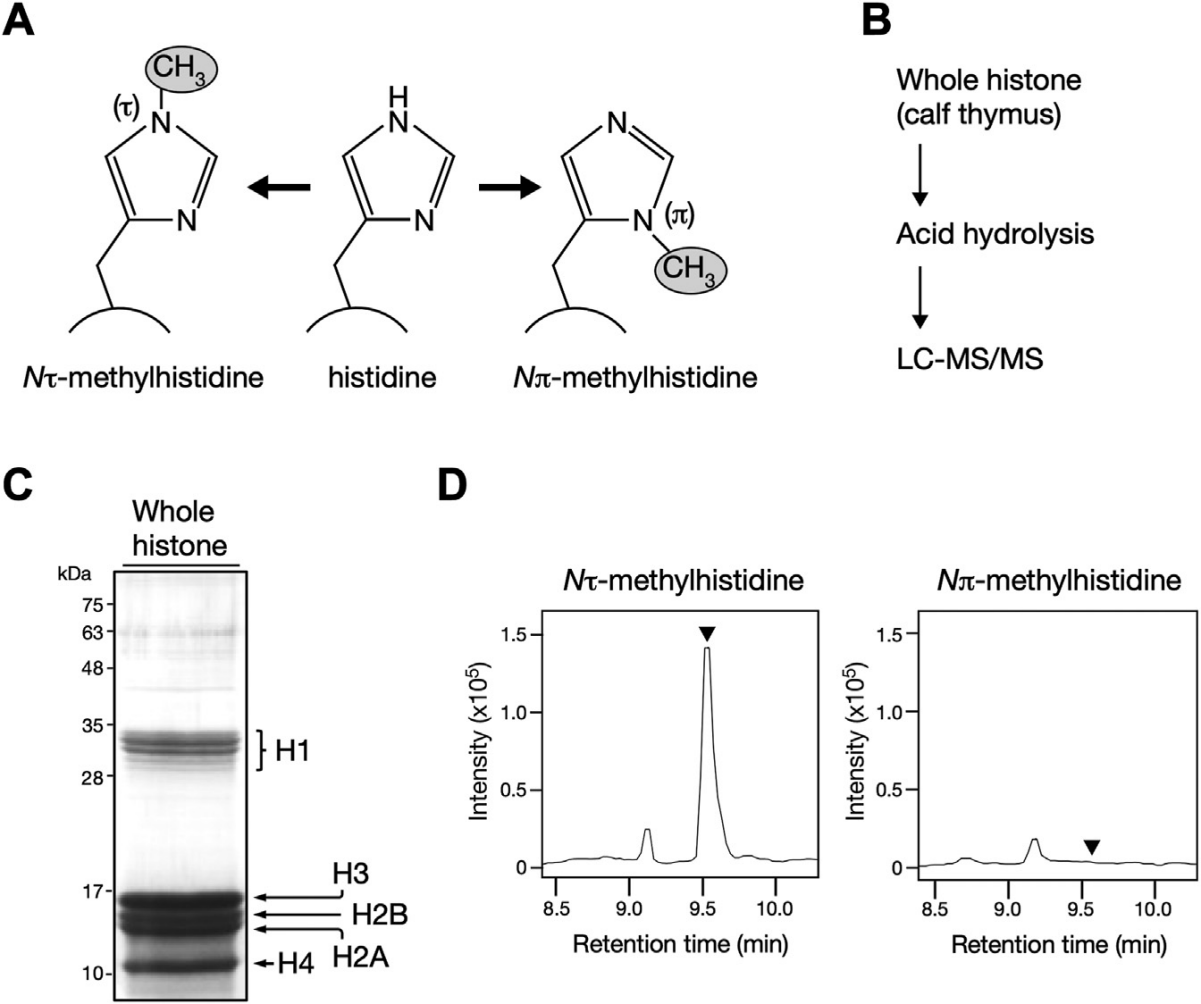
**Histidine *N*τ-methylation occurs in whole histone from calf thymus.***A*, histidine methylation yields two isomers of mono-methylhistidine. *Left*, *N*τ-methylhistidine, *Right*, *N*π-methylhistidine. The *circular arc* indicates the polypeptide backbone. *B*, schematic of biochemical strategy to determine which isomer of methylhistidine is included in whole histone from calf thymus. *C*, purity of whole histone from calf thymus. Whole histone was resolved using 18% SDS-PAGE followed by reverse staining. Core histone H2A, H2B, H3, and H4 are indicated with *arrows*. Linker histone H1 variants are indicated by a *bracket*. *D*, whole histone from calf thymus contains *N*τ-methylhistidine. Acid hydrolysate of whole histone was analyzed by LC-MS/MS, and its methylhistidine contents are shown as chromatograms. *Arrowheads* indicate the retention time of the *N*τ- and *N*π-methylhistidine defined by their standards. *Left*, *N*τ-methylhistidine (9.52 min); *right, N*π-methylhistidine (9.55 min).

The histidine residue can be monomethylated on either the distal (τ) or proximal (π) nitrogen atom of the imidazole ring, yielding two isomers: τMH or πMH (Fig. 1*A*). To determine whether histidine residue(s) of histone proteins are methylated, and if so, which isomer of methylhistidine is introduced, we adopted an approach that allows to distinguish between *N*τ and *N*π histidine methylation by detecting methylhistidine isomers after acid hydrolysis of the protein of interest (Fig. 1*B*). In addition, we employed commercially available histone proteins consisting of unfractionated whole histone from the calf thymus because they are a reproducible source often used for biochemical analysis (24, 25). It is important to note that the amino acid sequences of the core histones are nearly identical in mammalian species.

First, we checked the quality of the whole histone by reverse staining following SDS-PAGE and validated that core histones H2A, H2B, H3, and H4, and linker histone H1 were highly concentrated, with no apparent contamination (Fig. 1*C*). LC-MS/MS analysis revealed that the total protein hydrolysates of whole histone exhibited a strong peak at the retention time of τMH, whereas no peak was observed for πMH (Fig. 1*D*). These results indicated the presence of *N*τ-histidine methylation in whole histone from the calf thymus.

### Histidine Nτ-methylation is found in both core and linker histones

**Figure 2:**
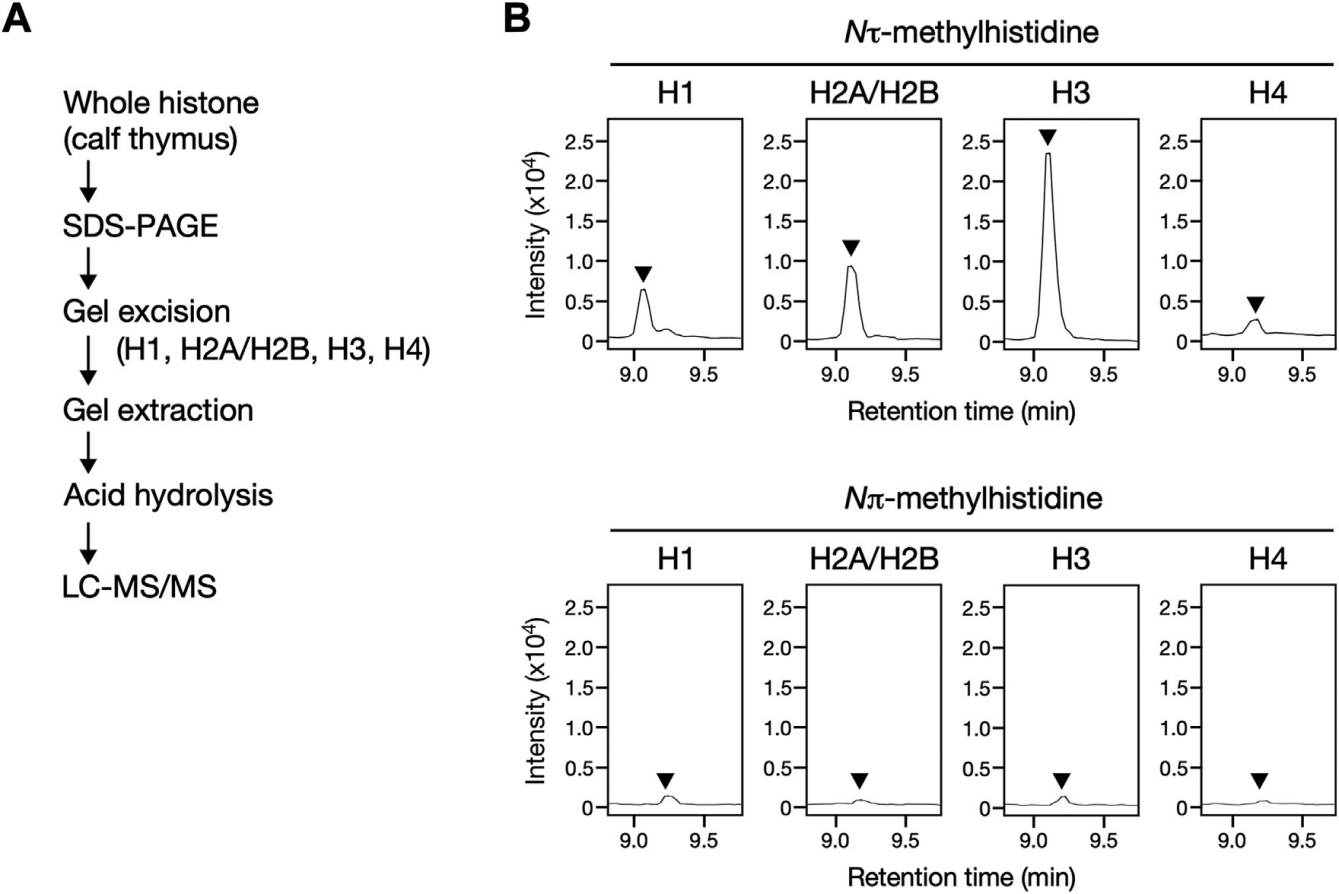
**Both core and linker histones contain *N*τ-methylhistidine.***A*, schematic of biochemical strategy to isolate each histone fraction and investigate their methylhistidine isomers. *B*, histidine *N*τ-methylation occurs in both core and linker histones from calf thymus. Acid hydrolysates of each histone fraction were analyzed by LC-MS/MS, and their methylhistidine contents are shown as chromatograms. *Arrowheads* indicate the retention time of the *N*τ- and *N*π-methylhistidine defined by their standards. *Top*, *N*τ-methylhistidine (9.11 min); *bottom*, *N*π-methylhistidine (9.22 min).

Since canonical core histones H2A, H2B, H3, and H4 and variants of the linker histone H1 contain several histidine residues in mammals, each of them could be potentially methylated. To further identify which histone proteins are the targets of histidine methylation, we attempted to isolate the linker histone H1 and the four core histones H2A, H2B, H3, and H4. Whole histone from calf thymus was resolved by SDS-PAGE and processed for reverse staining, as shown in Fig. 1*C*. Each band of H1, H3, and H4 was excised from the gel, and the proteins were extracted for amino acid analysis using LC-MS/MS (Fig. 2*A*). Meanwhile, because H2A and H2B could not be separated into distinct gel slices due to their approximately identical molecular weights, they were considered the same fraction, indicated as H2A/H2B. We found that fractions H1, H2A/H2B, and H3 contained substantial amounts of τMH and among them, with H3 being the most prominent (Fig. 2*B*, upper panel). In contrast, the H4 fraction exhibited a subtle peak compared to H1 and H2A/H2B (Fig. 2*B*, upper panel). Conversely, in agreement with the results from whole histone (Fig. 1*D*), no obvious peak of πMH was observed for each histone fraction (Fig. 2*B*, lower panel). These data suggested that *N*τ-histidine methylation occurred widely in both the core and linker histones, albeit at varying degrees.

### Histone H2A is monomethylated at His-82

**Figure 3:**
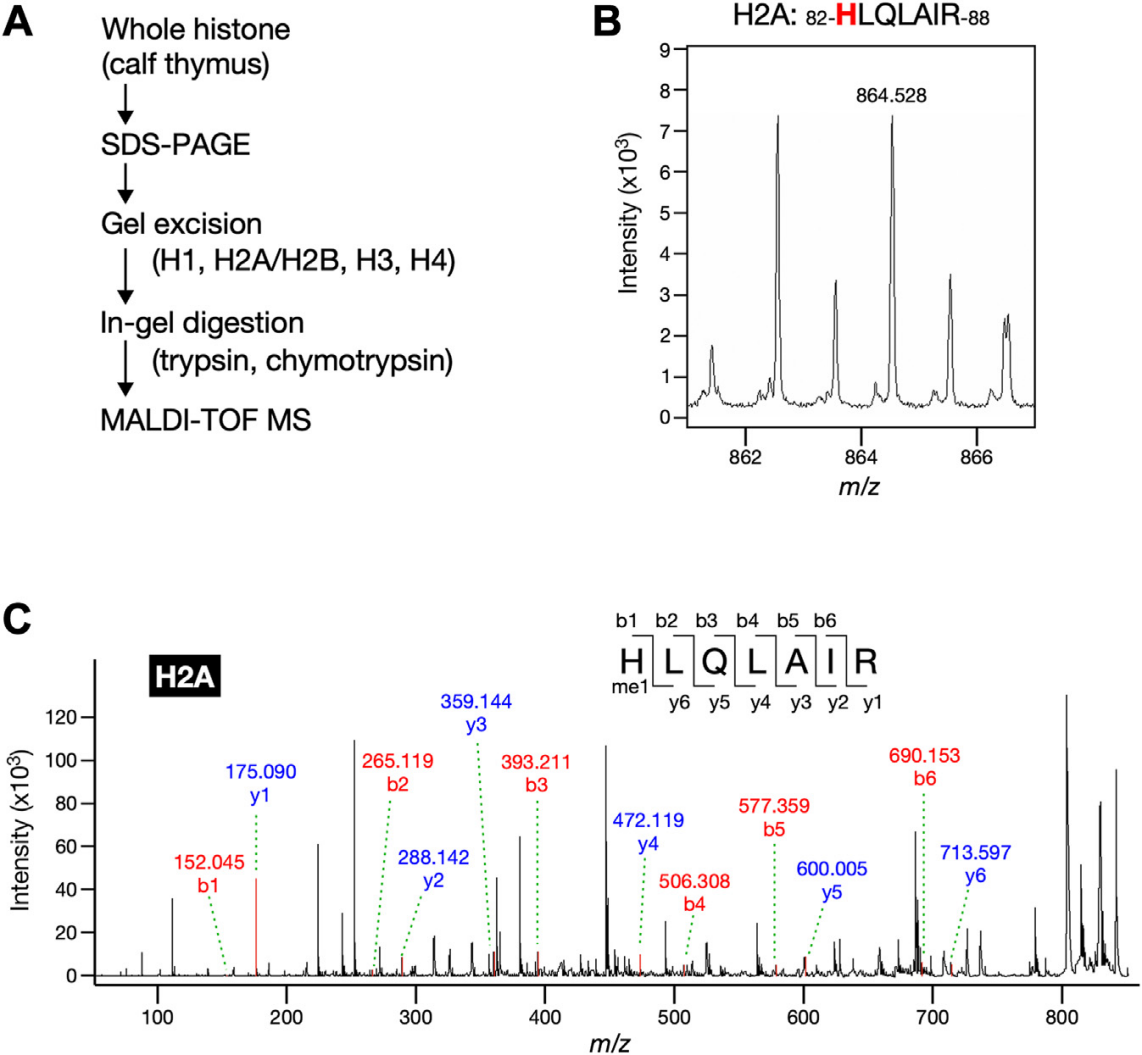
**Histone H2A from calf thymus is monomethylated at His-82.***A*, schematic of biochemical strategy to identify methylation sites of each histone fraction from calf thymus. *B*, the tryptic peptide of histone H2A contains monomethylated amino acid. Whole histone was resolved using 18% SDS-PAGE followed by reverse staining. The portion of gel corresponding to histone H2A and H2B fraction was digested with trypsin and then analyzed with MS. H2A-derived peptide is shown, with His-82 marked in *red*. *C*, MS/MS fragmentation spectra show monomethylation of histone H2A at His-82. The 7-amino acid peptide sequence and the b series (*red*) and y series (*blue*) peptide fragment ions for methylated peptide product are shown.

Next, we aimed to identify the histidine methylation sites in each histone. To this end, we digested histones H1, H2A/H2B, H3, and H4 in the excised gel with commonly used endopeptidases, such as trypsin and chymotrypsin, followed by MALDI-TOF MS analysis to explore methylated histone-derived peptides (Fig. 3*A*). No methylated peptides were detected in histones H1, H2B, H3, or H4. In contrast, histone H2A generated the peptide HLQLAIR (residues 82–88) that contained a monomethylated amino acid when digested with trypsin (Fig. 3*B*). Since this peptide contained histidine and arginine residues, both being potential targets for monomethylation, we further investigated the specific localization of the methyl group using MS/MS analysis. It was confirmed that His-82 of histone H2A was a monomethylation site (Fig. 3*C*). Collectively, these results demonstrated that histone H2A was methylated at least at His-82.

### Histone H3 is monomethylated at His-39

**Figure 4:**
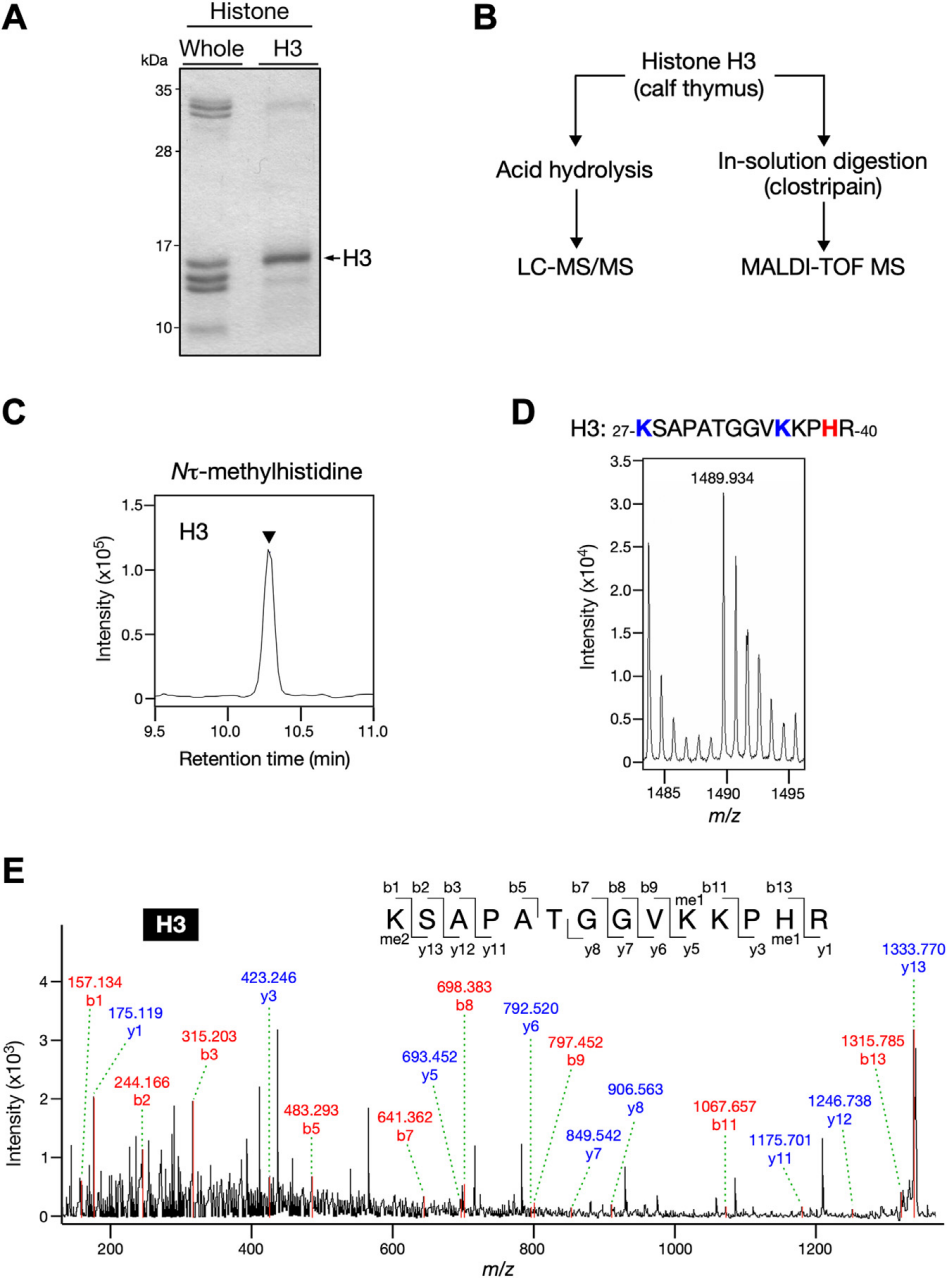
**Histone H3 from calf thymus is monomethylated at His-39.***A*, Purity of histone H3 from calf thymus. Whole histone and histone H3 fraction were resolved using 18% SDS-PAGE followed by Coomassie blue staining. Histone H3 is indicated by an *arrow*. *B*, schematic of biochemical strategies to determine which isomer of methylhistidine is included in histone H3 fraction (*left*) and to identify its methylation sites (*right*). *C*, histone H3 fraction from calf thymus contains *N*τ-methylhistidine. Acid hydrolysate of histone H3 fraction was analyzed by LC-MS/MS and its methylhistidine content is shown as a chromatogram. An arrowhead indicates the retention time of the *N*τ-methylhistidine defined by its standard (10.27 min). *D*, in-solution digestion of histone H3 with clostripain generates the peptide containing monomethylated amino acids. Histone H3 fraction was digested with clostripain and then analyzed using MS. H3-derived peptide is shown, with His-39 marked in *red*, Lys-27 and Lys-36 marked in *blue*. *E*, MS/MS fragmentation spectra show monomethylation of histone H3 at His-39. The 14-amino acid peptide sequence and the b series (*red*) and y series (*blue*) peptide fragment ions for methylated peptide product are shown.

Although histone H3 contained the highest amount of τMH (Fig. 2*B*), our attempt to detect H3 methylation by MALDI-TOF MS was initially unsuccessful (data not shown). The lysine- and arginine-rich sequences surrounding the putative methylation site His-39 (one of the two histidine residues in histone H3) might have been unfavorable for trypsin digestion. We therefore selected the endopeptidase clostripain that cleaves at the C terminus of arginine residues, which in turn generates the peptide KSAPATGGVKKPHR (residues 27–40), including His-39 of histone H3. Since clostripain is suitable for in-solution digestion, we obtained a commercially available histone H3 fraction from calf thymus, and its homogenous preparation was validated using SDS-PAGE followed by Coomassie blue staining (Fig. 4*A*).

First, we tested whether the histone H3 fraction contains τMH using acid hydrolysis and subsequent LC-MS/MS analysis (Fig. 4*B*). In accordance with the gel-excision results (Fig. 2*B*), the H3 fraction showed a strong peak at the retention time of τMH but not πMH (Fig. 4*C* and data not shown). Next, the H3 fraction was subjected to in-solution digestion with clostripain and analyzed using MALD-TOF MS (Fig. 4*B*). We successfully identified the peptide KSAPATGGVKKPHR (residues 27–40) to which four methyl groups could be added (Fig. 4*D*). Furthermore, to specifically identify the site in the peptide bearing the methyl group, we performed MS/MS analysis and found dimethylation at Lys-27 (H3K27me2), monomethylation at either Lys-36 (H3K36me1) or Lys-37 (H3K37me1), and monomethylation at His-39 (H3H39me1) (Fig. 4*E*). Importantly, this peptide includes H3K27me2 and H3K36me1, which are both established epigenetic marks. Taken together, these data suggested that *N*τ-histidine methylation occurred at least at the His-39 of histone H3.

### Histones H2A at His-82 and H3 at His-39 are monomethylated in cultured cells

**Figure 5:**
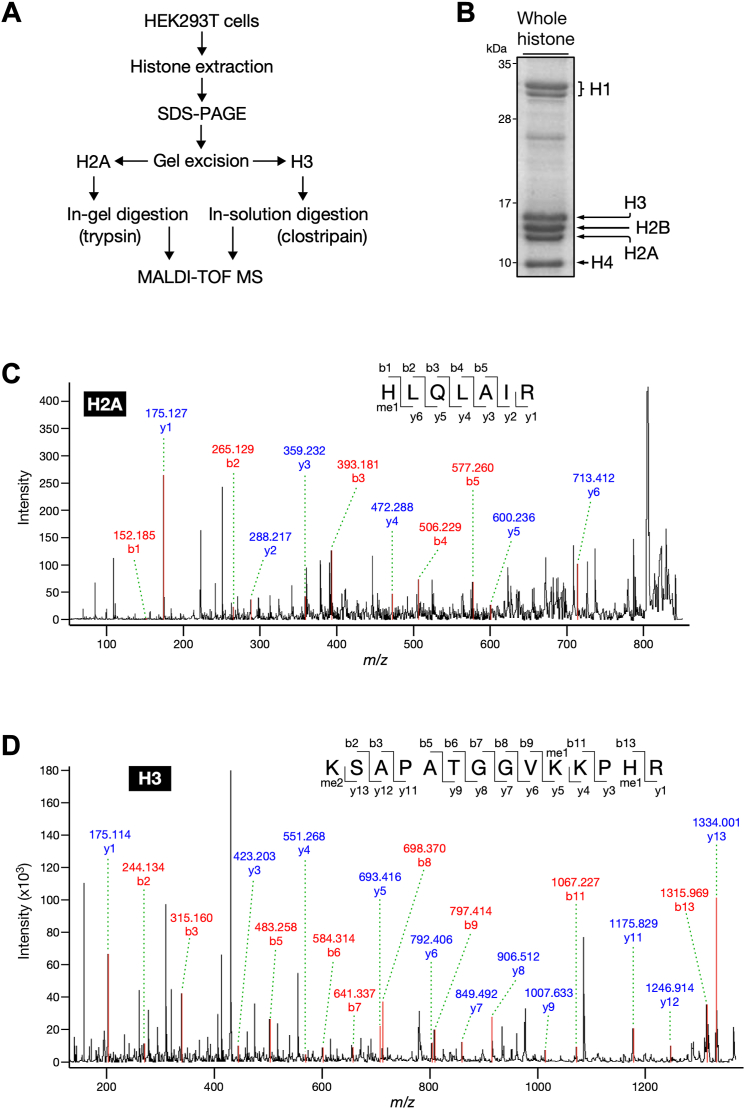
**Histones H2A and H3 from HEK293T cells are monomethylated at His-82 and His-39, respectively.***A*, schematic of biochemical strategy to identify methylation sites of histones H2A and H3 from HEK293T cells. *B*, purity of whole histone from HEK293T cells. Whole histone was resolved using 18% SDS-PAGE followed by Coomassie blue staining. Core histone H2A, H2B, H3, and H4 are indicated with *arrows*. Linker histone H1 variants are indicated by a *bracket*. The portion of gel corresponding to histone H2A fraction was digested with trypsin and then analyzed with MS. *C*, MS/MS fragmentation spectra show monomethylation of histone H2A at His-82. The 7-amino acid peptide sequence and the b series (*red*) and y series (*blue*) peptide fragment ions for methylated peptide product are shown. *D*, MS/MS fragmentation spectra show monomethylation of histone H3 at His-39. The 14-amino acid peptide sequence and the b series (*red*) and y series (*blue*) peptide fragment ions for methylated peptide product are shown.

**Figure S1:**
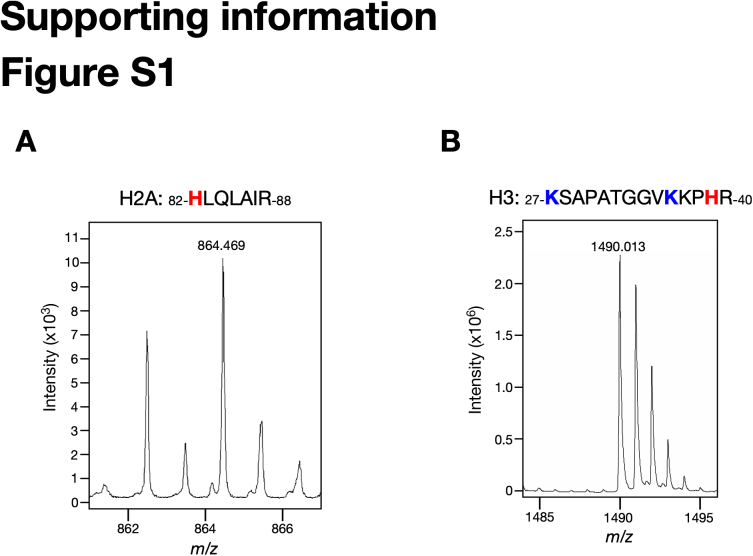
MS spectra of protease digests of histones H2A and H3 from HEK293T cells. *A*, the tryptic peptide of histone H2A contains monomethylated amino acid. Whole histone form HEK293T cells was resolved using 18% SDS-PAGE followed by Coomassie blue staining. The portion of gel corresponding to histone H2A was digested with trypsin and then analyzed with MS. H2A-derived peptide is shown, with His-82 marked in *red*. *B*, in-solution digestion of histone H3 with clostripain generates the peptide containing monomethylated amino acids. Whole histone form HEK293T cells was resolved using 18% SDS-PAGE followed by reverse staining. The portion of gel corresponding to histone H3 was destained and then eluted from the gel. After digestion with clostripain, the peptides were analyzed using MS. H3-derived peptide is shown, with His-39 marked in red, Lys-27 and Lys-36 marked in *blue*.

Based on the above results obtained from calf thymus histones, we optimized the suitable conditions for detecting histidine methylation in histones. Subsequently, we addressed the question of whether histones in human cells are also methylated at histidine residues by using a human cell line HEK293T. The whole histone fraction was purified from the cells by acid extraction and then separated using SDS-PAGE followed by in-gel trypsin digestion of histone H2A and in-solution clostripain digestion of histone H3 (Fig. 5, *A* and *B*). MALDI-TOF MS analysis demonstrated that histones H2A and H3 generated the peptides HLQLAIR (residues 82–88) and KSAPATGGVKKPHR (residues 27–40), respectively, both of which had almost identical mass-to-charge ratios to those derived from calf thymus histones (Fig. S1, *A* and *B*). Furthermore, MS/MS spectra of these peptides showed that histone H2A at His-82 and histone H3 at His-39 were monomethylated, coincident with the data using calf thymus histones (Fig. 5, *C* and *D*). These results reinforced the notion that histone histidine methylation occurs widely in mammalian cells.

### Histone H3 methylation occurs mainly in lysine and to a lesser extent in arginine and histidine

Finally, to estimate the overall methylation status of histone H3, we measured the total amount of methylated amino acids, including methyllysine, methylarginine, and methylhistidine, using the acid hydrolysates of calf thymus histone H3. The extent of methylation was described as the ratio of the amount of methylated amino acids to that of total amino acids (*e.g.*, τMH/total histidine). As shown in Table 1, histone H3 methylation occurred primarily in lysine, and from among its three distinct forms, dimethyllysine was higher than monomethyllysine, and trimethyllysine (12.13% *versus* 9.60% and 5.87%, respectively). Unlike lysine methylation, the extent of arginine and histidine methylation was substantially low (0.05% and 0.32%, respectively). In particularly, although the ratio of methylarginine appeared to be subtle, it could be attributed to the arginine-rich sequence of histone H3; namely, the numbers of arginine and histidine residues are 18 and 2, respectively, in the canonical histone H3. Considering that arginine methylation has so far been reported to occur at Arg-2, Arg-8, Arg-17, Arg-26, and Arg-42 out of the 18 arginine residues, the extent of arginine methylation tends to be lower than that of histidine (26, 27, 28, 29, 30, 31, 32). Collectively, these data uncovered that the methylation of histone H3 was markedly concentrated at lysine residues, while it was only clearly detectable at the arginine and histidine residues.

**Table 1:** Overall methylation status of histone H3 from calf thymus

Amino acid	Methyl amino acid	Methylation content
Lys	MML	9.60 ± 0.81%
	DML	12.13 ± 2.47%
	TML	5.87 ± 0.87%
Arg	MMA	0.05 ± 0.02%
	ADMA	0.05 ± 0.01%
	SDMA	0.05 ± 0.03%
His	τMH	0.32 ± 0.04%
	πMH	0.00 ± 0.00%

Acid hydrolysates of calf thymus histone H3 were analyzed by LC-MS/MS and various methyl amino acid contents are shown as the ratio of the amount of methylated amino acid to that of total amino acid. Mean ± s.d. (*n* = 3 independent experiments). MML, monomethyllysine; DML, dimethyllysine; TML, trimethyllysine; MMA, monomethylarginine; ADMA, asymmetric dimethylarginine; SDMA, symmetric dimethylarginine; τMH, *N*τ-methylhistidine; πMH, *N*π-methylhistidine.

## Discussion

In this study, we demonstrated that histones H2AH82 and H3H39 underwent *N*τ-methylation in mammals. Two early seminal papers had reported the presence of histidine methylation in the histone fraction. In 1969, Gershey *et al.* labeled the cellular proteins of mature and immature avian erythrocytes with L-methionine-(methyl-^3^H) as the methyl group donor and found that histone fractions I (lysine-rich) and V (serine-rich) contained radioactive 3-methylhistidine (21). In 1972, Borun *et al.* performed pulse labeling of HeLa S3 cells with both L-[I-^14^C]-methionine and L[methyl-^3^H]-methionine and estimated that histidine methylation occurred to the same extent as arginine methylation in the histone f3 fraction (22). However, both these studies raised the open question of which histone(s) and which histidine residue(s) were the targets of methylation. The methylation site(s) have long been unidentified in histone proteins because of the following two possibilities (1): the use of unpurified fractions that may contain actin or other cellular histidine-methylated proteins and (2) technological limitations at that time (20). After half a century, the combination of modern MS/MS with highly purified histones has revealed that histidine *N*τ-methylation occurs, at least in histones H2AH82 and H3H39. Further investigations on the remaining methylation sites are required to clarify the role of histidine methylation in histone proteins.

What is the biological significance of histidine *N*τ-methylation in histones H2AH82 and H3H39? According to the tertiary structure of human nucleosome (RCSB ID: 3AFA), the His-82 residue of histone H2A is located in the globular domain and positioned to interact with the surrounding amino acid residues *via* hydrogen bonding (33). In contrast, the His-39 residue of histone H3 is located at the disordered tail domain and also close to proximity to Lys-36 (H3K36), which can be modified by mono-, di-, or tri-methylation (H3K36me1, H3K36me2, and H3K36me3, respectively) (34). Histone H3K36 methylation is a well-known hallmark of active transcription, where H3K36me2 is preferentially enriched proximal to transcription start sites (35). Furthermore, SETD2, the sole enzyme responsible for H3K36me3 in metazoans, is recruited by elongating RNA polymerase II depositing H3K36me3 in the gene bodies of active genes (36). Intriguingly, crystal structure analysis of the SETD2/histone H3 complex revealed that the SETD2 catalytic domain interacts with the H3 peptide at Lys-36 and its flanking residues from Ala-29 to Arg-42 *via* hydrogen bonding (37). This raised the possibility that H3H39 methylation may affect H3 recognition by SETD2, consequently decreasing the H3K36me3 levels. ChIP-sequencing data with an anti-H3H39me antibody will allow us to determine whether H3K36me3 and H3H39me are mutually exclusive in active genes.

Histone H2A can be functionally and structurally classified into the two domains, the flexible N- or C-terminal “tails” that are extended from the nucleosome-associated structure and the globular parts located within the nucleosome, respectively (38). The two domains are targets of multiple posttranslational modifications, such as acetylation, phosphorylation, ubiquitination, and methylation (39). These posttranslational modifications on the tails are linked to the alteration of chromatin status by using acetyl, phosphorus, ubiquitin, or methyl groups as chemical flags that can recruit “reader proteins” of the so called “histone code” (5). While the functional contribution of histone tails as “deciphering machineries” is well investigated so far, the globular domains have long been thought as platforms for the formation of nucleosome. However, despite the limited information regarding the methylation located in the globular domains, lysine methylation of histone H3 at Lys-79 in yeast was identified by advanced, more sensitive mass spectrometric techniques, which was shown to play a pivotal role in telomeric silencing (40). In addition, the methylation of glutamine located in the globular domain of histone H2A at Gln-105 in yeast and at Gln-104 in human cells was also identified, suggesting its modes of action as an RNA polymerase-I–dedicated modification (41). Therefore, our finding of the histidine methylation of histone H2A at His-82 in the globular domain will give us basic and important information to consider its diverse and biological significance and to screen a modification enzyme.

It is not known what an enzyme catalyzes histidine *N*τ-methylation of histone proteins. In mammals, only three enzymes, SETD3, METTL18, and METTL9, have been shown to be histidine-specific methyltransferase, and the former two enzymes introduce τMH in each specific substrate, namely cytoskeleton actin and ribosomal protein RPL3, respectively (15, 16, 18, 19, 42, 43). Unlike the arginine methyltransferase family, histidine methyltransferases do not comprise a group of closely related paralogs (44). Therefore, we preliminarily tested whether these enzymes were responsible for histone methylation by siRNA knockdown in HEK293T cells, but LC-MS/MS analysis of cellular histone fractions demonstrated that there was no significant decrease in the amount of πMH, even when SETD3, METTL18, or METTL9 were depleted (data not shown). Although whole histone fractions extracted from cultured cells contain more contaminants than those extracted from the calf thymus owing to the fractionation process, it appears likely that other histidine methyltransferases are involved in histone methylation. Considering possible uniqueness of histidine methyltransferases, it is also conceivable that the histone methylation of histidine residues might be catalyzed by a new or atypical family of enzymes and/or a requirement of cofactors that remain to be identified.

We found that methylation of histone H3 occurs mainly in lysine and to a lesser extent in arginine and histidine (Table 1). Given the distribution of lysine methylation in chromatin where H3K4 and H3K36 are enriched in euchromatic regions, while H3K9me and K27me are concentrated in heterochromatic regions (45), it appears to account for the higher extent of lysine methylation in histone H3. In contrast, the arginine methylation level was very low, although H3R2, H3R8, H3R17, H3R26, and H3R42 are targets of a family of protein arginine methyltransferases (46). Notably, this result is consistent with the previous report over 50 years ago in which Paik and Kim determined the amount of methylarginine in various cellular fractions and showed that methylarginine is hardly detectable in histone fractions from the calf thymus (47). In contrast, interestingly, they also revealed that histone fractions isolated from rat liver nuclei contain a relatively large amount of methylarginine (47). These findings imply that histone arginine methylation levels may vary in different cell types, and the same idea could be applied to histidine methylation. Establishing antibodies detecting endogenous histone H3H39 methylation would enable us to investigate its distribution in different cell types and alterations in methylation levels when exposed to environmental stimuli.

Our unique LC-MS/MS approach, using acid hydrolysates of whole histone, disclosed the overall methylation status of histone H3 (Table 1). This quantitative method is simple, useful, and advantageous for examining the types and degrees of methylation of lysine, arginine, and histidine residues in the proteins of interest (48). However, since the other posttranslational modifications, such as phosphorylation of serine, threonine, and tyrosine residues and acetylation of lysine residues, are removed under acid hydrolysis conditions (6 M HCl at 110 °C for 24 h), it cannot be applied to understand the overall histone modifications including acetylation and phosphorylation. Moreover, considering that histones are the components of the nucleosome subunit, information on their genomic location as well as methylation context is also required to understand the biological roles of histone histidine methylation in transcriptional regulation. Methodological advances in this field will shed light on the significance of histone histidine methylation and establish it as a new cipher that add a layer of complexity to the histone code.

Note: As mentioned above, histidine residues can potentially be monomethylated at either *N*τ or *N*π positions. Thus, in accordance with histone arginine dimethylation, denoted as H3R2me2a (asymmetric dimethylation) and H3R2me2s (symmetric dimethylation), we proposed that histone histidine *N*τ-monomethylation should be denoted as H2AH82me1t and H3H39me1t, respectively.

## Experimental procedures

### Reagents

Histone proteins were purchased from the following commercial sources: unfractionated whole histone from calf thymus (Sigma-Aldrich; H9250) and histone H3 from calf thymus (Roche; 11034758001). Proteases qualified for use in mass spectrometry sample preparation are as follows: trypsin (Promega; V5111), chymotrypsin (Promega; V1061), and clostripain (Promega; V1881). Standards used in LC-MS/MS are as follows: L-lysine monohydrochloride (Sigma-Aldrich; L-5626), *N*ε-monomethyl-L-lysine hydrochloride (Sigma-Aldrich; 04,685), *N*ε,*N*ε -dimethyl-L-lysine monohydrochloride (Sigma-Aldrich; 19,773), *N*ε*,N*ε*,N*ε*-*trimethyl-lysine hydrochloride (Sigma-Aldrich; T1660), L-arginine hydrochloride (Arg, Sigma-Aldrich; A-5131), *N*G-monomethyl-L-arginine monoacetate salt (Sigma-Aldrich; 475,886), *N*G,*N*G-dimethylarginine dihydrochloride (Sigma-Aldrich; D4268), *N*G*,N′*G*-*dimethyl-L-arginine dihydrochloride (Santa Cruz; sc-222070), L-histidine hydrochloride monohydrate (His, Wako; 084–00702), 3-methyl-L-histidine (Sigma-Aldrich; M9005), 1-methyl-L-histidine (Sigma-Aldrich; 67,520), *N*ω-propyl-L-arginine hydrochloride (Wako; 512–27111).

### Analysis of methyl amino acids of histone proteins by LC-MS/MS

The acid-hydrolyzed (6 M HCl at 110 °C for 24 h) histone samples were dissolved in 10 μl of water and injected into a Shimadzu Nexera X2 ultra-high pressure liquid chromatography - LCMS-8050TM triple quadrupole mass spectrometer system. SeQuant ZIC-HILIC column (2.1 × 150 mm, 3.5 μm; Merck KGaA) with a SeQuant ZIC-HILIC Guard Fitting (1.0 × 14 mm; Merck KGaA) was applied for chromatographic separation. The chromatographic conditions were described previously (18). As the internal standard, 25 ng of *N*-propyl-L-arginine (N-PLA) were spiked into the histone samples. All the solvents used in this work were analytical reagent grade.

MS/MS analysis was performed by collision-induced dissociation on precursor ions, and the resulting product ions were selectively detected (multiple-reaction monitoring: MRM). Ion intensities were drawn over time, and mass chromatograms were obtained. The optimized instrumental parameters were also described previously (18). The transitions based on the MRMs of lysine, monomethyllysine, dimethyllysine, and trimethyllysine were determined as m/z 147.10 > 84.20, 161.20 > 84.15, 175.15 > 84.15, and 189.15 > 84.15, respectively. As described above, the MRMs of arginine, monomethylarginine, asymmetric dimethylarginine, symmetric dimethylarginine, and N-PLA were m/z 175.0 > 70.05, 189.15 > 70.15, 203.15 > 158.05, 203.15 > 171.95, and 217.3 > 70.15, respectively. The MRMs of histidine, πMH and τMH were determined as m/z 156.1 > 110.1, 170.1 > 96.1, and 170.1 > 124.1, respectively.

The chromatographic and mass spectrometric system operation, data acquisition, and processing were carried out using the LabSolutions for LC–MS, version 5.60 software (Shimadzu). Standard amino acid derivatives and N-PLA were dilution series in the range of 0.1 to 10 pmol. The calibration curve was generated by plotting the peak area ratios of analyte to the internal standards against concentration using a linear regression. Then, concentrations and percentage of analytes observed in each experiment were calculated from calibration curves.

### Purification of each histone protein by SDS-PAGE

Whole histones from calf thymus or HEK293T cells were separated by 18% SDS-PAGE under the conditions of 200 V and 90 min, followed by negatively stained with EzStainReverse (ATTO, AE-1310). The band corresponding to each histone subunit was excised, and the gel slices were destained with 25 mM Tris-glycine pH 8.0. The gel bands were crushed to yield gel microparticles (gel slurry) (49). Histone proteins were passively eluted from the gel slurry using 25 mM Tris-glycine, pH 8.0. After filtration of gel pieces by ATTO Prep MF cartridges (ATTO, 3521370), histone proteins were finally purified from the eluate by methanol–chloroform extraction.

### Enzymatic digestion for MALDI-TOF MS

After separation of whole histone by SDS-PAGE, bands corresponding each subunit were excised and digested in gel with trypsin and chymotrypsin after reductively alkylation treatment with DTT and iodoacetamide. After 24 h of incubation at 37 °C in incubation buffer (50 mM NH_4_HCO_3_ and 1 mM CaCl_2_), digested peptides were eluted from the polyacrylamide gel by sonication in acetonitrile containing 0.1% formic acid.

For in-solution digestion of histone H3, the lyophilized powder of histone H3 from calf thymus (Roche) or the gel-purified histone H3 from HEK293T cells were dissolved in incubation buffer (50 mM Tris-HCl pH7.8, 5 mM CaCl_2_ and 2 mM EDTA) in the presence of clostripain. After adding 10 × activation buffer (50 mM Tris-HCl pH7.8, 50 mM DTT and 2 mM EDTA), the reactions were incubated at 37 °C for 4 h.

### Identification of histone histidine methylated sites by MALDI-TOF MS

The protease-digestive peptides were desalted with ZipTip μC_18_ (Millipore; ZTC18S096). Saturation solution of R-cyano-4-hydroxycinnamic acid (Shimadzu GLC) in 10 mg/ml in 0.1% trifluoroacetic acid and 50% acetonitrile was used as the MALDI matrix. MS and MS/MS analysis were carried out by ultrafleXtreme NTA (Bruker) under CID conditions. The mass spectrometer was operated in the positive-ion mode and reflector mode under high voltage conditions as follows: Ion Source1: 25.00 kV, Ion Source2: 22.40 kV, Lens: 11.85 kV, Reflector: 26.45 kV, and Reflector2: 13.45 kV. The MS/MS spectra were acquired using LIFT method under high voltage conditions as follows: Ion Source1: 7.50 kV, Ion Source2: 6.75 kV, Lens: 3.50 kV, Reflector: 29.50 kV, Reflector2: 14.00 kV, LIFT1: 19.00 kV, and LIFT2: 4.10 kV. The acquired data were processed by FlexAnalysis, version 3.3, (Bruker) and BioTools, version 3.2, (Bruker) software. To identify histone proteins, Mascot, version 2.8.2, (Matrix Science) was utilized as an auxiliary tool, and then, UP9136_B_taurus was used as the reference database. Analysis of methylated residues was carried out by RapiDeNovo in BioTools Version 3.2 (Bruker).

### Histone extraction from cultured cells

HEK293T cells were cultured in DMEM (Nacalai Tesque) supplemented with 10% FBS (Gibco) and 1% Penicillin-Streptomycin solution (Sigma-Aldrich). Cells were washed with PBS (−) and then lysed at a cell density of 1 × 10^7^ cells per ml in ice-cold lysis buffer [100 mM Hepes pH7.1, 150 mM NaCl, 1.5 mM MgCl_2_, 0.5% NP-40; freshly added with Protease Inhibitor Cocktail (Nacalai Tesque)] for 15 min on a rotator at 4 °C. Thereafter, the cells were centrifuged at 1,500*g* for 10 min at 4 °C, and the supernatant was discarded. This step was followed by a washing step of the pellet with lysis buffer and another centrifugation to remove the residual soluble proteins. The pellet was resuspended in 0.4 M H_2_SO_4_ and then centrifuged at 1,500*g* for 15 min at 4 °C. The supernatant containing histones was mixed with 10 times the volume of 100% acetone and precipitated overnight at −20 °C. Histones were collected by centrifuge at 1,500*g* for 10 min and rinsed with 90% acetone. The pellet was air-dried and dissolved with water for SDS-PAGE analysis.

## Data availability

The MS data have been deposited in the jPOSTrepo under ID: JPST002114 and JPST002184. Some unpublished data available upon request from the corresponding author, email: hiroakid@tara.tsukuba.ac.jp, on reasonable request.

## Supporting information

This article contains supporting information.

## Conflict of interest

The authors declare no conflict of interest with the contents of this article.
